# Effects of green-manure and tillage management on soil microbial community composition, nutrients and tree growth in a walnut orchard

**DOI:** 10.1038/s41598-021-96472-8

**Published:** 2021-08-19

**Authors:** Ningguang Dong, Guanglong Hu, Yunqi Zhang, Jianxun Qi, Yonghao Chen, Yanbin Hao

**Affiliations:** Beijing Academy of Forestry and Pomology Sciences, Beijing Academy of Agriculture and Forestry Sciences, Beijing, 100093 People’s Republic of China

**Keywords:** Agroecology, Microbial ecology

## Abstract

This study characterized the effect of green manures (February orchid, hairy vetch, rattail fescue and a no-green-manure control) and the termination method (flail or disk) on nutrient contents, enzyme activities, microbial biomass, microbial community structure of rhizosphere soil and vegetative growth of walnut tree. All three selected green manures significantly enhanced the water content, organic C, total N and available P. The rattail fescue significantly decreased the mineral N. Total organic C, total N, mineral N and available P were significantly greater under flail than under disk. Hairy vetch and February orchid significantly improved levels of soil β-glucosidase, N-acetyl-glucosaminidase and acid phosphatase activity, whereas rattail fescue improved only β-glucosidase activity. All of the green manures significantly decreased phenoloxidase activity. β-glucosidase, N-acetyl-glucosaminidase and acid phosphatase activities were significantly greater under flail relative to disk. The termination method had no significant effect on phenoloxidase activity. The different types of green manures and termination methods significantly altered the soil microbial biomass and microbial community structure. The green-manure treatments were characterized by a significantly greater abundance of Gram-positive (Gram +) bacteria, total bacteria and saprophytic fungi compared to the control. Hairy vetch significantly decreased the abundance of arbuscular mycorrhizal fungi (AMF) while February orchid and rattail fescue increased their abundance compared to the no-green-manure treatment. The abundance rates of Gram+ bacteria, actinomycetes, saprophytic fungi and AMF were significantly greater in soils under flail than under disk. In terms of vegetative growth of walnut tree, hairy vetch showed the greatest positive effects. The growth of walnut tree was significantly greater under flail relative to disk. Our results indicate that green-manure application benefits the rhizosphere soil micro-ecology, rhizosphere soil nutrient contents and tree growth. Overall, the hairy vetch and flail combined treatment is recommended for walnut orchards in northern China.

## Introduction

Walnut (*Juglans regia*) is cultivated for its edible nuts and timber and is one of the most valuable and widely cultivated horticultural commodities in the world. Walnuts rank only behind almonds in tree nut production, and China leads the world production of walnuts^1^. With the rapid development of the walnut industry in China, intensive management practices such as weeding, tillage and excessive use of chemical fertilizers and pesticides have resulted in degraded soil health, environmental pollution and decreased biodiversity. In recent years, organic farming has received a considerable amount of public attention due to its suggested benefits to food safety and its benign effects on the environment. Application of organic amendments, such as green manure, crop residues and livestock manure, is one of the most economic and effective sustainable agriculture practices to improve soil organic matter (SOM) content and crop yield^2, 3^.

The use of green manure provides plants with nutrients and therefore stimulates soil microbiological activities. Organic matter supplied from green-manure and plant residues affects soil moisture, temperature, and pH, inducing short- and medium-term responses in microbial populations^4^. The species composition of green manure plays an important role in determining the potential benefits to the soil following incorporation. For example, green manure enriched in leguminous species is associated with increased soil nitrogen (N) content, likely due to N-fixation, and it therefore changes microbial community structure and activity^5^. Microorganisms are key players in important ecological processes, with soil microorganisms regulating the main processes that occur in soil such as cycling of biogeochemicals, promoting plant growth, decomposing organic matter and various interactions with physical soil processes^6^. Soil microbial diversity is also crucial in maintaining soil health^7^. Different microbial communities are responsible for specific functions during the decomposition of green manures and plant residues. Accordingly, soil microbial parameters are key indicators of changes in soil health under different soil-management practices, due to their high sensitivity to environmental changes and tight correlation with the soil and ecosystem^6, 8^.

Conservation-agriculture-based practices (e.g. zero tillage, green manure and residue retention) can lead to significant changes in the biochemical properties of soils, alter the activities of the soil microbial community and improve soil health^9, 10^. Several studies have shown that reduced tillage, green manure and N-fertilization result in changes in soil microbial structure and biochemical properties^4, 5, 11–13^. Tillage generally shifts soil microbial communities toward aerobic species with the high metabolic rates typical of bacterial species. Previous studies found that switching from a no-tillage system to a disk or plough management system reduced the ratios of fungi to bacteria^14, 15^. While general soil disturbances often cause predictable changes in microbial community composition, there have been some reports that different tillage practices (e.g. disk, mouldboard plough or chisel plough) have differing effects on microbial populations. For example, Drijber et al.^16^ demonstrated distinct community differences between a sub-plough undercutter and a mouldboard plough tillage system.

February orchid (*Orychophragmus violaceus* L.), hairy vetch (*Vicia villosa* Roth.) and rattail fescue (*Vulpia myuros* L.) are commonly used for green manure in walnut orchards in northern China. These green manures have been used in organic production systems for years and have adapted to the local climate and soil conditions. However, their influences on the soil micro-ecological environment, soil health and the tree growth remain unknown. Additionally, optimization of green-manure species and the termination method is necessary.

In this study, a 3-yr field experiment was conducted using a combination of two factors: green manures (February orchid, hairy vetch and rattail fescue) and two termination methods (flail or disk). A treatment without green manure was applied as a control. The effects of these treatments on soil nutrient contents, enzyme activities, soil microbial biomass, microbial community structure and tree growth were investigated. We hypothesized that green manures and the termination method (flail or disk) can change soil microbial communities associated with the rhizosphere of walnut. Subsequently, these effects generate an increase in microbial biomass, soil enzyme activity, and nutrient cycling, which as a consequence, stimulates walnut growth. The objectives of this study were to use the soil micro-ecological environment and tree growth to determine the benefits of specific green-manure management combinations and to provide references for the selection of the optimal green-manure type and termination method for walnut orchards.

## Results

### Soil chemical properties

As shown in Table 1, tillage and green manure had no significant effect on soil pH. The water contents in the February orchid, hairy vetch and rattail fescue treatments were 26.9%, 26.3% and 11.5% higher than in the no-green-manure treatment, respectively. Soil water contents were significantly greater under flail relative to disk (*P* < 0.05), independent of the species used for the green manure. Soil TOC was significantly higher under the green-manure treatment, with February orchid, hairy vetch and rattail fescue treatments being 22.9%, 24.6% and 12.7% higher than the no-green-manure treatment, respectively. The same trend was observed for TN, with February orchid, hairy vetch, and rattail fescue showing increases of 12.4%, 49.4% and 10.1%, respectively. Tillage had a significant effect on TOC and TN, as the flail treatments had significantly greater levels compared to disk (*P* < 0.05). The flail treatments of hairy vetch and February orchid had greater soil TOC content than other treatment combinations. Soil TN in the hairy-flail treatment combination was higher than other treatment combinations.

**Table 1:** Selected properties of walnut orchard soils (0–20 cm) as affected by green-manure and termination method.

Treatment**t**	pH	Water content %	Organic C g kg^−1^	Total N g kg^−1^	Mineral N mg kg^−1^	Available P mg kg^−1^	Available K mg kg^−1^
**GM**							
FO	8.11 ± 0.04a	19.8 ± 0.7bc	14.5 ± 0.4bc	1.0 ± 0.05de	34.2 ± 1.8de	54.4 ± 3.2def	346 ± 16cde
HV	8.15 ± 0.06 a	19.7 ± 0.6bc	14.7 ± 0.7abc	1.33 ± 0.13ab	40.7 ± 2.2ab	83 ± 4.8ab	291 ± 11f.
RF	8.12 ± 0.04 a	17.4 ± 0.5f.	13.3 ± 0.4d	0.98 ± 0.04de	29.4 ± 1.6fg	44.2 ± 2.9gh	384 ± 20ab
NGM	8.13 ± 0.06 a	15.6 ± 0.4g	11.8 ± 0.2f.	0.89 ± 0.01g	36.4 ± 1.6cd	33.7 ± 2.5i	195 ± 9g
**Tillage**							
Disk	8.13 ± 0.05 a	17.8 ± 0.5ef	13.2 ± 0.4de	1.02 ± 0.05d	32.3 ± 1.5ef	55 ± 3.4de	339 ± 15e
Flail	8.12 ± 0.04 a	20.1 ± 0.7abc	15.0 ± 0.6abc	1.18 ± 0.09bc	37.2 ± 2.1bcd	66.1 ± 3.8c	341 ± 16de
**GM** × **Tillage**							
FO and disk	8.08 ± 0.03 a	18.1 ± 0.5def	13.2 ± 0.2d	0.92 ± 0.01f	31.3 ± 1.5ef	49.6 ± 3.1efg	351 ± 15bcde
FO and flail	8.13 ± 0.05 a	21.4 ± 0.8a	15.7 ± 0.5a	1.07 ± 0.08cd	37.1 ± 2.0bcd	59.2 ± 3.3cd	340 ± 16e
HV and disk	8.16 ± 0.07 a	18.9 ± 0.5cd	13.8 ± 0.6cd	1.20 ± 0.11abc	38.4 ± 1.8bc	75.3 ± 4.4b	286 ± 10f.
HV and flail	8.13 ± 0.04 a	20.4 ± 0.7ab	15.5 ± 0.8ab	1.45 ± 0.14a	42.9 ± 2.5a	90.7 ± 5.1a	295 ± 12f.
RF and disk	8.15 ± 0.04 a	16.3 ± 0.4g	12.6 ± 0.3e	0.94 ± 0.02ef	27.2 ± 1.2g	40.1 ± 2.8h	379 ± 19abc
RF and flail	8.09 ± 0.03a	18.5 ± 0.5de	13.9 ± 0.5cd	1.02 ± 0.05d	31.6 ± 1.9ef	48.3 ± 2.9fg	388 ± 21a

The values are means ± standard deviations (n = 3). Values followed by different letters differ significantly (LSD test, *P* < 0.05). The *P* values of ANOVA are shown in Table S1. *GM* green manure; *FO* February orchid; *HV* hairy vetch; *RF* rattail fescue; *NGM* no green manure.

The hairy vetch treatment distinctly increased the soil mineral nitrogen level and the rattail fescue treatment significantly reduced the soil mineral nitrogen content compared with the no-green-manure treatment (*P* < 0.05), whereas the February orchid treatment had no significant effect on soil mineral nitrogen content (*P* > 0.05). Soil mineral nitrogen was significantly greater under the flail relative to the disk (*P* < 0.05). The hairy vetch-flail treatment combination had significantly greater soil mineral nitrogen content than other treatment combinations (*P* < 0.05). Soil AP was 1.3–2.5 times higher and soil AK was 1.5–2.0 times higher with the green-manure treatments than the no-green-manure treatment (*P* < 0.05). Significant differences in soil AP and AK contents were observed among the three green-manure treatments (*P* < 0.05), where the hairy vetch had the highest AP content and the rattail fescue had the highest AK content. Tillage had no significant effect on soil AK (*P* > 0.05), while soil AP was significantly greater under flail relative to disk (*P* < 0.05). Soil AP content was highest in the hairy vetch-flail treatment combination.

### Soil enzyme activities

**Figure 1:**
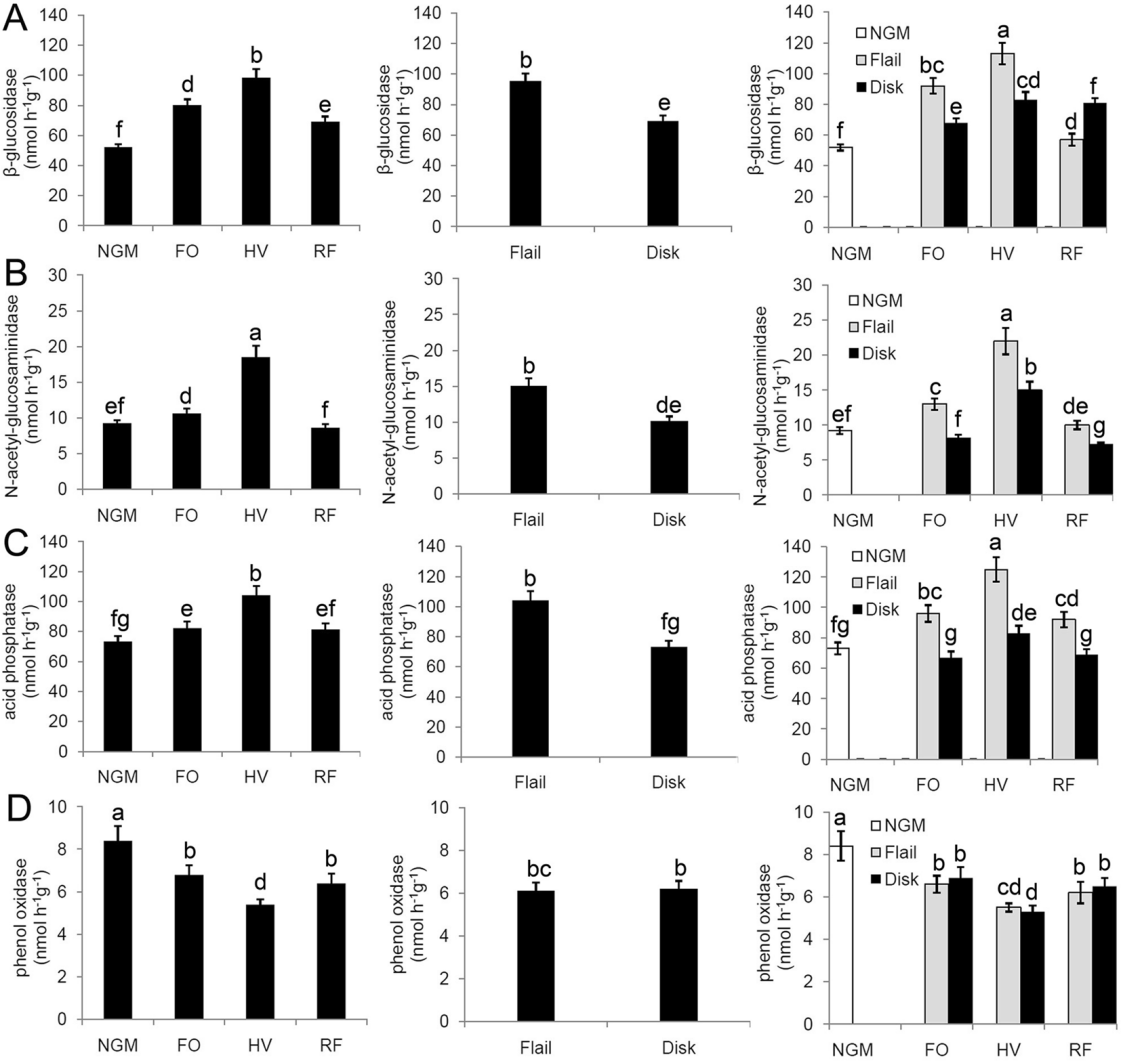
Soil enzyme activities in response to the green-manure treatment termination method. (**A**) β-glucosidase, (**B**) N-acetyl-glucosaminidase, (**C**) acid phosphatase and (**D**) phenoloxidase. The *P* values of ANOVA are shown in Table S2. Bars with different letters differ significantly according to the LSD test (*P* < 0.05). *GM* Green manure; *FO* February orchid; *HV* hairy vetch; *RF* rattail fescue; *NGM* no green manure. The histogram was created using Microsoft Excel 2010 (Microsoft, Redmond, Washington, USA).

The potential soil metabolic capacity of the microbial community, as determined by selected enzyme activities, significantly differed based on the green-manure and tillage types (Fig. 1). Soil β-glucosidase activity was significantly higher under the green-manure treatment, with the February orchid, hairy vetch, and rattail fescue treatments being 53.8%, 88.5% and 32.7% higher than the no-green-manure treatment, respectively (Fig. 1A). Soil N-acetyl-glucosaminidase and acid phosphatase enzyme activities were significantly higher in the February orchid and hairy vetch treatments compared to the no-green-manure treatment (Fig. 1B,C) (*P* < 0.05). Rattail fescue had no significant effect on soil N-acetyl-glucosaminidase or acid phosphatase enzyme activities (Fig. 1B,C) (*P* > 0.05). Significant differences in N-acetyl-glucosaminidase and acid phosphatase enzyme activities were observed among the three green-manure treatments, in which the hairy vetch treatment had the highest N-acetyl-glucosaminidase and acid phosphatase activities (*P* < 0.05). All green-manure treatments revealed significant decreases in soil phenoloxidase activity (Fig. 1D).

The soil β-glucosidase, N-acetyl-glucosaminidase and acid phosphatase activities were also affected by the termination method. The enzyme activities were significantly greater under flail relative to disk with β-glucosidase, N-acetyl-glucosaminidase and acid phosphatase having 37.7%, 48.5% and 42.5% higher activity (Fig. 1A–C). However, the termination method had no significant effect on phenoloxidase activity (Fig. 1D) (*P* > 0.05). Additionally, Soil β-glucosidase, N-acetyl-glucosaminidase and acid phosphatase enzyme activities were significantly higher in the hairy vetch-flail treatment combination compared to other treatment combinations.

### Soil microbial biomass

**Figure 2:**
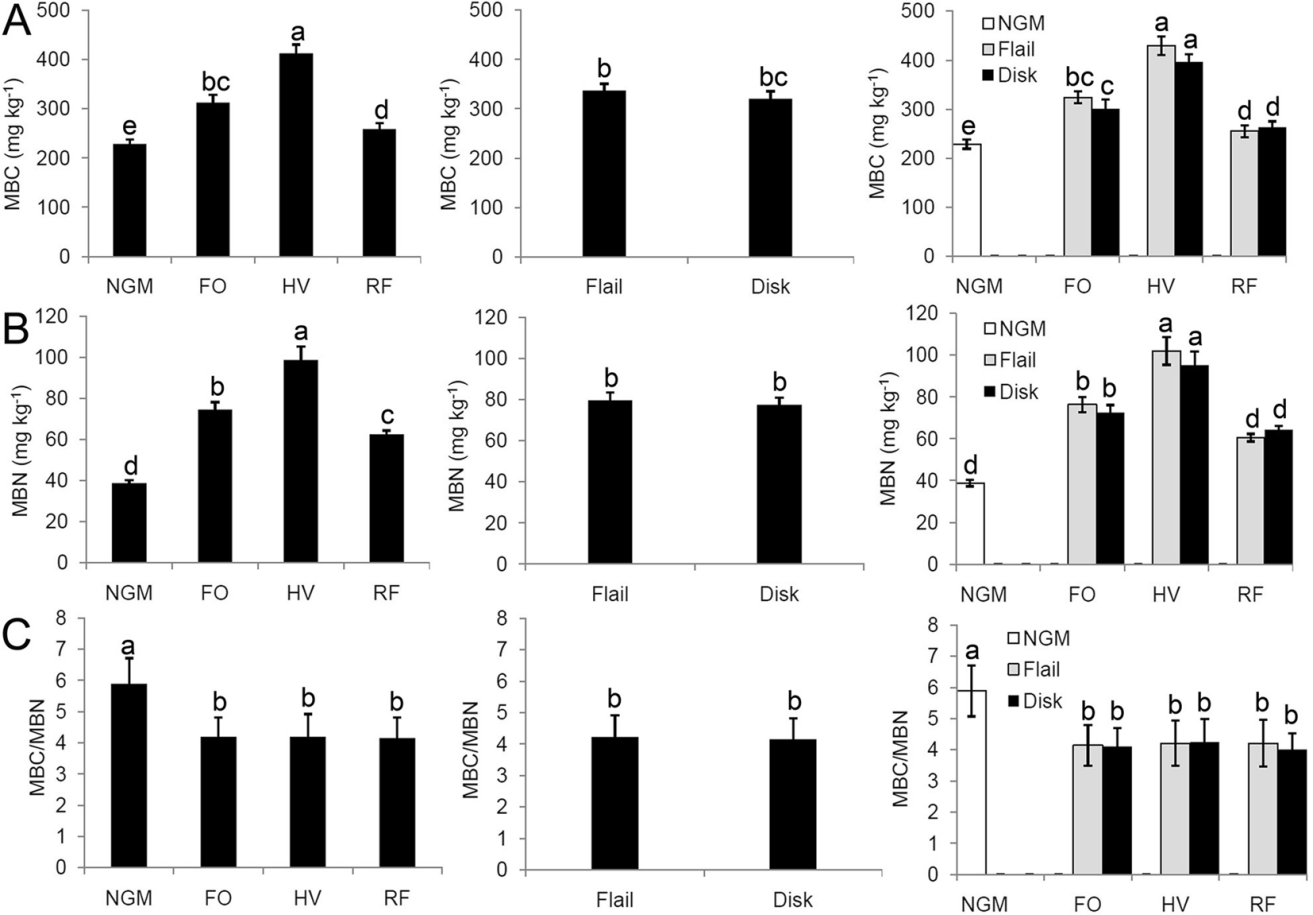
Soil microbial biomass as affected by both the green-manure treatment and termination method. (**A**) Microbial biomass carbon (MBC), (**B**) microbial biomass nitrogen (MBN) and (**C**) MBC/MBN. Bars with different letters differ significantly according to the LSD test (*P* < 0.05). The *P* values of ANOVA are shown in Table S3. *GM* Green manure; *FO* February orchid; *HV* hairy vetch; *RF* rattail fescue; *NGM* no green manure. The histogram was created using Microsoft Excel 2010 (Microsoft, Redmond, Washington, USA).

MBC was significantly higher under the green-manure treatments, with February orchid, hairy vetch and rattail fescue treatments being 36.7%, 80.7% and 13.3% higher than the no-green-manure treatment, respectively (Fig. 2A). The same trend was observed for MBN, with February orchid, hairy vetch and rattail fescue showing increases of 91.9%, 154.1% and 60.9%, respectively (Fig. 2B). All green-manure treatments significantly decreased the soil MBC/N (Fig. 2C) (*P* < 0.05). However, no difference in soil MBC/N was observed among the three green-manure treatments (Fig. 2C) (*P* > 0.05). Tillage did not have a significant influence on MBC, MBN or MBC/N (Fig. 2) (*P* > 0.05).

### Soil microbial community structure

The microbial community structures estimated by the FAME profiles are shown in Table 2. Green manure had the greatest effect on total FAMEs in the February orchid and hairy vetch treatments compared to rattail fescue and the no-green-manure treatments (*P* < 0.05). The microbial community structure composition was significantly shifted by the green-manure treatments (Table 2). The abundance of FAME biomarkers for Gram+ bacteria (sum of i15:0, a15:0, i16:0, i17:0 and a17:0) and the total bacteria (sum of Gram+ bacteria, Gram− bacteria and actinomycetes) were significantly greater in the green-manure treatments than in the no-green-manure treatment (*P* < 0.05). However, no differences in the abundance of Gram+ bacteria or total bacteria were observed among the three green-manure treatments (*P* > 0.05). No significant difference was observed in the abundance of Gram− (sum of 16:1ω7c, 16:1ω9c, cy17:0 and cy19:0) and actinomycetes (sum of 10Me16:0, 10Me17:0 and 10Me18:0) between the green-manure treatments and the no-green-manure treatment (*P* > 0.05). Green manure treatments resulted in a significantly greater relative abundance of the saprophytic fungi biomarker (sum of 18:1ω9c, 18:2ω6c, 18:3ω6c) than the no-green-manure treatment (*P* < 0.05). Hairy vetch significantly decreased the relative abundance of the AMF biomarker (16:1ω5c), while February orchid and rattail fescue had a significantly greater abundance in the AMF compared to the no-green-manure treatment (*P* < 0.05).

**Table 2:** Microbial community composition according to FAME profiles as affected by green-manure and the termination method.

Treatment	Total FAME	bacterial			Total bacteria	Fungi		Total fungi	G+/G-	F/B
		G+	G-	actinomycetes		saprophytic	AMF			
**GM**										
FO	73.89 ± 3.58ab	11.24 ± 0.35ab	8.40 ± 0.27a	4.89 ± 0.11ab	24.53 ± 1.09a	8.24 ± 0.34ab	1.45 ± 0.05b	9.69 ± 0.36abc	1.32 ± 0.04ab	0.4 ± 0.01bcd
HV	76.78 ± 3.55ab	10.94 ± 0.36bc	8.23 ± 0.26a	4.78 ± 0.12abc	23.95 ± 1.13a	8.24 ± 0.31ab	1.22 ± 0.04e	9.46 ± 0.35bcd	1.33 ± 0.04a	0.39 ± 0.01
RF	61.94 ± 3.29c	10.84 ± 0.36bc	8.20 ± 0.24a	4.72 ± 0.11bc	23.76 ± 1.01a	8.32 ± 0.36ab	1.46 ± 0.05b	9.77 ± 0.40abc	1.32 ± 0.04ab	0.41 ± 0.01abc
NGM	59.32 ± 3.22c	8.87 ± 0.30d	8.19 ± 0.24a	4.70 ± 0.11bc	21.76 ± 0.97b	7.57 ± 0.28c	1.35 ± 0.04c	8.92 ± 0.31d	1.08 ± 0.03d	0.41 ± 0.01abc
**Tillage**										
Disk	70.44 ± 3.46b	10.62 ± 0.34bc	8.31 ± 0.25a	4.66 ± 0.11c	23.59 ± 1.06ab	7.82 ± 0.31bc	1.26 ± 0.04de	9.07 ± 0.35cd	1.28 ± 0.03c	0.38 ± 0.01d
Flail	71.29 ± 3.71ab	11.39 ± 0.41a	8.25 ± 0.26a	4.91 ± 0.12ab	24.55 ± 1.16a	8.71 ± 0.37a	1.49 ± 0.05ab	10.2 ± 0.41ab	1.38 ± 0.05a	0.42 ± 0.01ab
**GM × Tillage**										
FO and disk	72.46 ± 3.25ab	10.85 ± 0.33bc	8.38 ± 0.26a	4.75 ± 0.11bc	23.98 ± 1.02a	7.81 ± 0.33bc	1.31 ± 0.04cd	9.12 ± 0.35cd	1.29 ± 0.03bc	0.38 ± 0.01d
FO and flail	75.31 ± 4.07ab	11.63 ± 0.38a	8.42 ± 0.28a	5.02 ± 0.12a	25.07 ± 1.15a	8.67 ± 0.35a	1.58 ± 0.06a	10.25 ± 0.37a	1.38 ± 0.05a	0.41 ± 0.01abc
HV and disk	77.57 ± 3.63a	10.55 ± 0.34c	8.31 ± 0.25a	4.62 ± 0.11c	23.48 ± 1.08ab	7.79 ± 0.28bc	1.12 ± 0.03f.	8.91 ± 0.30d	1.27 ± 0.03c	0.38 ± 0.01d
HV and flail	75.99 ± 3.51ab	11.33 ± 0.42a	8.20 ± 0.26a	4.89 ± 0.12ab	24.42 ± 1.18a	8.69 ± 0.34a	1.31 ± 0.04cd	10 ± 0.36ab	1.38 ± 0.05a	0.41 ± 0.01abc
RF and disk	61.29 ± 3.10c	10.46 ± 0.34c	8.25 ± 0.25a	4.60 ± 0.11c	23.31 ± 0.98ab	7.85 ± 0.35bc	1.34 ± 0.05cd	9.19 ± 0.38cd	1.27 ± 0.03c	0.39 ± 0.01cd
RF and flail	62.58 ± 3.38c	11.22 ± 0.40ab	8.14 ± 0.23a	4.83 ± 0.11ab	24.19 ± 1.05a	8.78 ± 0.39a	1.57 ± 0.05a	10.35 ± 0.42a	1.38 ± 0.05a	0.43 ± 0.01a

Small letters within a column reflect differences between treatments (LSD test, *P* < 0.05). The *P* values of ANOVA are shown in Table S4. *G*+ gram positive bacteria, *G − *gram negative bacteria, *AMF* arbuscular mycorrhizal fungi, *G*+*/G − *gram positive bacteria: gram negative bacteria ratio, *F/B* fungi: bacteria ratio, *GM* green manure; *FO* February orchid; *HV* hairy vetch; *RF* rattail fescue; *NGM* no green manure.

Total FAMEs were not significantly altered by tillage (*P* > 0.05). However, tillage significantly affected the community structure composition. The abundance of FAME biomarkers for Gram+ bacteria, actinomycetes, saprophytic fungi and AMF fungi were significantly greater in soils under flail than under disk (*P* < 0.05). Additionally, flail resulted in significantly greater ratios of Gram+: Gram− and fungi: bacteria FAME biomarkers compared to disk (*P* < 0.05). No significant difference in the abundance of Gram− and total bacteria was observed between flail and disk (*P* > 0.05).

**Figure 3:**
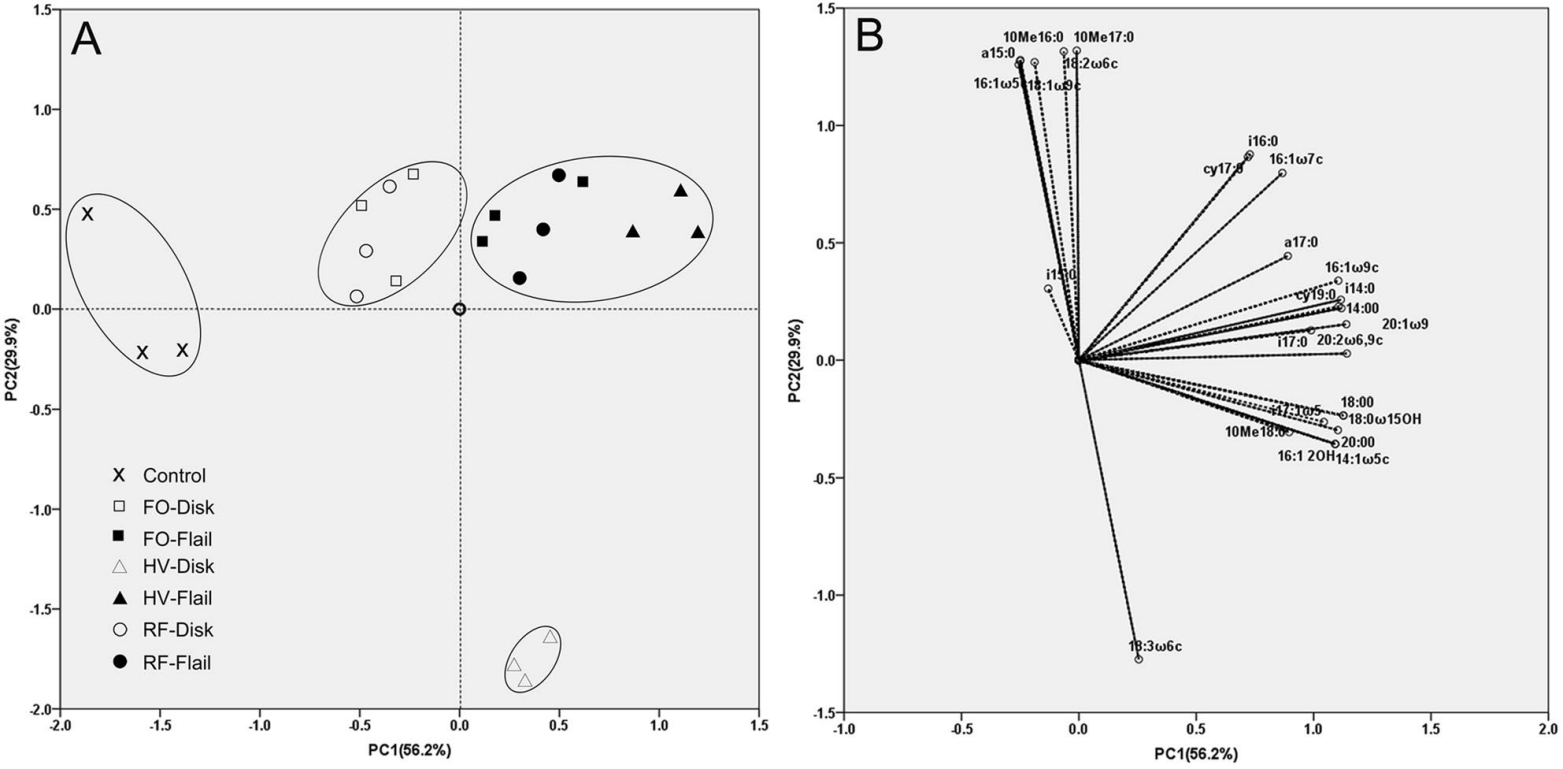
Principal components analysis (PCA) of fatty acid methyl esters (FAMEs) (**A**) and graph of variables (**B**) for individual FAMEs from a PCA of the green-manure and termination-method treatments. The 2D plot was generated by Canoco 4.5 (MicrocomputerPower, Ithaca, USA).

Differentiation of the microbial community structure was detected based on the PCA analysis of 26 FAME biomarkers consistently present in all samples (Fig. 3). PC1 and PC2 explained 56.2% and 29.9% of the overall variance, respectively. A clear separation between the no-green-manure control treatment and all other treatment groups was observed along PC1 (Fig. 3A). The February orchid and rattail fescue treatments were separated from the hairy vetch treatment when combined with disk for termination along PC1 (Fig. 3A). Both of flail-green manure and disk-green manure treatments were separately from the control along PC1 (Fig. 3A). Along PC1, the termination method was effective in separating the treatments within February orchid and rattail fescue, but not within hairy vetch (Fig. 3A). The most obvious separation occurred between the hairy vetch-disk treatment and all other treatment groups along PC2 (Fig. 3A). The PC loadings for individual FAMEs are shown in Fig. 3B. Communities under the February orchid-flail, hairy vetch-flail, and rattail fescue-flail treatments were associated with a greater relative abundance of Gram+ bacteria (i16:0, i17:0, a17:0) and Gram− bacteria (16:1ω7c, 16:1ω9c, cy17:0, cy19:0). Communities under the February orchid-disk and rattail fescue-disk treatments enhanced the proportion of actinomycetes (10Me16:0, 10Me17:0) and saprophytic fungi (18:1ω9c, 18:2ω6c) and AMF (16:1ω5c). The community under the hairy vetch-disk treatment was associated with a high relative abundance of actinomycetes (10Me18:0) and saprophytic fungi (18:3ω6c).

### Vegetative growth of walnut

As shown in Table 3, in terms of tree height, ground diameter and crown breadth, hairy vetch treatment had the highest value, followed by February orchid, rattail fescue and no-green-manure treatment. Tree height and ground diameter were significantly higher in the February orchid and hairy vetch treatments compared to the no-green-manure treatment (Table 3) (*P* < 0.05). However, no significant differences were observed in the tree height or ground diameter between the rattail fescue and no-green-manure treatments (Table 3) (*P* > 0.05). Crown breadth was significantly higher under the green-manure treatment, with the February orchid, hairy vetch, and rattail fescue treatments being 25.2%, 37.6% and 23.2% higher than the no-green-manure treatment, respectively (Table 3) (*P* < 0.05). The tree height, ground diameter and crown breadth were also affected by the termination method. The tree growth was significantly greater under flail relative to disk with tree height, ground diameter and crown breadth having 17.0%, 18.0% and 14.5% higher values (Table 3) (*P* < 0.05).

**Table 3:** Tree height, ground diameter and crown breadth of walnut tree as affected by green manure and termination method.

Treatment	Tree height m	Ground diameter cm	Crown breadth cm
**GM**			
FO	4.96 ± 0.23bcd	7.73 ± 0.37bc	447.16 ± 18cd
HV	5.16 ± 0.26abc	8.53 ± 0.45ab	491.43 ± 22a
RF	4.58 ± 0.21def	7.28 ± 0.31cd	440.24 ± 16cd
NGM	4.22 ± 0.18f.	6.88 ± 0.21de	357.20 ± 13f.
**Tillage**			
Disk	4.50 ± 0.21ef	7.23 ± 0.33cde	428.55 ± 17de
Flail	5.31 ± 0.26ab	8.46 ± 0.47ab	490.67 ± 23ab
**GM** × **Tillage**			
FO-Disk	4.59 ± 0.21def	7.19 ± 0.31cde	415.97 ± 15de
FO-Flail	5.37 ± 0.25ab	8.27 ± 0.49ab	478.35 ± 23abc
HV-Disk	4.73 ± 0.22cde	7.83 ± 0.39bc	465.81 ± 19bc
HV-Flail	5.59 ± 0.28a	9.23 ± 0.52a	517.05 ± 24a
RF-Disk	4.19 ± 0.19f.	6.68 ± 0.24e	403.87 ± 12e
RF-Flail	4.97 ± 0.25bcd	7.88 ± 0.38bc	476.61 ± 23abc

The values are means ± standard deviations (n = 3). Values followed by different letters differ significantly (LSD test, *P* < 0.05). The *P* values of ANOVA are shown in Table S5. *GM* green manure; *FO* february orchid; *HV* hairy vetch; *RF* rattail fescue; *NGM* no green manure.

Table 4 showed that compared with the walnut trees in the no-green-manure treatment, the trees in the green manure treatments were found to have an increasing trend in root length, root length density and root surface area. Root length under the February orchid, hairy vetch, and rattail fescue treatments was 1453, 1477 and 1357 cm respectively, being 1.05 times, 1.04 times and 1.01 times the length of no-green-manure treatment. Compared to no-green-manure treatment, root length density under February orchid, hairy vetch, and rattail fescue treatments were 16.5%, 23.7% and 9.0% higher respectively. Root surface area under February orchid, hairy vetch, and rattail fescue treatments were 12.5%, 17.5% and 6.9% higher than the no-green-manure treatment, respectively. Tillage had a significant effect on root length, root length density and root surface area, as the flail treatments had significantly greater levels compared to disk (Table 4) (*P* < 0.05). Root length, root length density and root surface area were 12.8%, 17.2% and 14.1% higher under flail relative to disk, respectively.

**Table 4:** Root length, root length density and root surface area of walnut tree as affected by green manure and termination method.

Treatment	Root length cm	Root length density cm cm^−3^	Root surface area cm^2^
**GM**			
FO	1453 ± 51bcde	71.3 ± 5.4abc	521.7 ± 19.8bc
HV	1477 ± 56abcd	75.7 ± 4.1ab	544.8 ± 21.2ab
RF	1357 ± 49efg	66.7 ± 3.0cd	495.6 ± 14.9cde
NGM	1335 ± 45fg	61.2 ± 2.8de	463.7 ± 19.4ef
**Tillage**			
Disk	1343 ± 47fg	65.6 ± 3.6cde	486.3 ± 15.3de
Flail	1515 ± 59abc	76.9 ± 5.1ab	555.1 ± 23.5ab
**GM** × **Tillage**			
FO-Disk	1345 ± 49fg	66.4 ± 3.9cde	485.3 ± 16.7de
FO-Flail	1561 ± 61ab	76.2 ± 5.6ab	558.1 ± 23.6ab
HV-Disk	1386 ± 47defg	69.8 ± 3.8bc	518.9 ± 18.9bcd
HV-Flail	1568 ± 58a	81.6 ± 5.2a	570.7 ± 26.1a
RF-Disk	1298 ± 46g	60.6 ± 2.8e	454.7 ± 12.8f.
RF-Flail	1416 ± 53cdef	72.8 ± 3.9abc	536.5 ± 20.5ab

The values are means ± standard deviations (n = 3). Values followed by different letters differ significantly (LSD test, *P* < 0.05). The *P* values of ANOVA are shown in Table S6. *GM* green manure; *FO* February orchid; *HV* hairy vetch; *RF* rattail fescue; *NGM* No green manure.

## Discussion

Green manure species and termination method have been deemed as critical factors in improving soil fertility and crop growth. This study examined the combined effects of green manure and termination method on soil nutrient contents, enzyme activities, soil microbial community composition and tree growth in a walnut orchard. Our results supported the hypothesis that green manures and the termination method can change soil microbial communities associated with the rhizosphere of walnut and then affect walnut growth. This work confirmed that the hairy vetch and flail treatment combination is an optimal agricultural practice for walnut orchards in northern China.

In this study, green manure increased soil moisture (Table 1) because the green manure reduced evaporation as compared to the no-green-manure treatment, a benefit that has been reported^17^. The green manures were used to cover the bases of trees or were turned into the soil, thereby contributing to the soil nutrient resources via microbial decomposition. Walnut orchards have a low amount organic residue returned to the soil since nearly the whole aboveground walnut biomass is exported. In the present study, increased soil C and N contents were found in all green-manure treatments (Table 1), indicating that green manure compensates for the SOC and N losses associated with the growth and development of walnut tree. Similar results were also reported by Nivelle et al.^4^ and Verzeaux et al.^18^ that cover crop can compensate for such organic outputs and improve soil nutrient contents. The February orchid and hairy vetch treatments significantly increased SOC compared to the rattail fescue treatment, indicating that such benefits may highly depending on green manure species utilized. Soil TN was significantly higher in the leguminous green-manure (hairy vetch) treatment compared to the other treatments, due to the nitrogen fixing capacity of hairy vetch. Our results showed that all three types of green manures significantly increased soil AP and AK (Table 1), indicating an active effect of green manures on soil P and K. The hairy vetch treatment distinctly increased soil mineral nitrogen levels, whereas they were significantly lower in the rattail fescue treatment than in the no-green-manure treatment. These results suggest nitrogen competition between the rattail fescue and the walnut trees. In contrast, leguminous green manure increases nitrogen input into soil due to its capacity to fix atmospheric N^17, 19^.

The soil-enzyme activities of the microbial community can be used as an indicator of soil organic matter decomposition potential and nutrient availability^2^. β-glucosidase, N-acetyl-glucosaminidase and acid phosphatase are involved in the cycling of carbon, nitrogen and phosphorus, and oxidative enzymes are responsible for degrading recalcitrant compounds like lignin and aromatic herbicides. The present study showed that soil β-glucosidase activity was significantly higher in the green-manure treatment compared to the no-green-manure treatment (Fig. 1A), in agreement with earlier studies^10, 20^. This may have been attributed to the higher level of soil organic C found in the plots under a green manure treatment. In agreement, Li et al.^21^ and Xu et al.^22^ observed a strong positive relationship between soil organic matter and soil β-glucosidase activity. Although the February orchid and hairy vetch increased the N-acetyl-glucosaminidase and acid phosphatase enzyme activities, the rattail fescue did not (Fig. 1B,C), when compared to the no-green-manure treatment. This finding indicates that different types of green manure have different effects on N-acetyl-glucosaminidase and acid phosphatase enzyme activities. N-acetyl-glucosaminidase is responsible for the acquisition of N by degrading chitin, and high soil N levels induced its increase to acquire N in this study. The increase in acid phosphatase activity under the February orchid and hairy vetch implies greater decomposition of organic P and that more P was available than in the control treatment, which is consistent with our result that the February orchid and hairy vetch treatments had higher P contents compared to the rattail fescue and no-green-manure treatments. As acid phosphatase activity plays a significant role in P bioavailability from native organic P compounds, it is frequently used as an indicator of P availability in soils^6^.

In this study, the soil POX activity showed a different pattern compared to the soil hydrolase activities, where the highest POX activity was achieved in the control (Fig. 1D). Factors known to influence phenoloxidase in soils include the concentration of SOM, the lignin content of plant litter, soil pH, and nitrogen availability^23, 24^. The green manure treatments significantly increased SOM compared to the control. Evidence suggests that high POX activity limits SOM accumulation^2, 24^. Therefore, decreased POX activity was observed in green manure treatment relative to the control in this study. Additionally, phenoloxidase is responsible for lignin degradation and mitigating the toxicity of aromatic compounds^24^. There is no difference in recalcitrant aromatic pesticides between green manure treatments and control since only eco-friendly pesticides were applied to the walnut orchards. The major difference between green manure treatments and control is the lignin content of plant litter with green manure treatments having higher lignin content. Our results showed POX activity was lower in green manure treatment relative to the control in spite of having a greater lignin content of plant litter. Phenoloxidase is produced primarily by fungi^7, 23, 24^. Fungi generally play a major role in initial degradation of plant litter, and lignin particularly, but most of the solubilized products of lignin degradation are probably metabolized by bacteria^24^. A few bacteria can degrade lignin using dehydrogenases^25^.

SMB is often used as an indicator of soil health and included in certain soil health indices^5^. In our study, there was an evident MBC increase in response to green manure in comparison with the control (Fig. 2A), which supports the FAME results (Table 2). Soil microbes are typically C-limited and lower microbial biomass in soils can be explained by low SOC^26^. Green manures provide more organic matter, which facilitates rapid microbial population growth. However, Liang et al.^27^ reported no change in the microbial biomass after using green manures, indicating that sampling time may be inappropriate for correlating microbial biomass response to applications of green manure.

The various green manures release specific compounds into the soil, leading to changes in the composition of the microbial communities associated with the type of green manure employed. This study demonstrated that the use of any green manure increased total FAMEs and altered the microbial community structure. This finding was reflected by the PCA (Fig. 3), which separated the controls from the rest of the treatments and revealed differences in FAME composition. In this study, the abundances of FAME biomarkers for total bacteria and Gram+ bacteria were significantly greater in the green-manure treatments than in the no-green-manure treatment (Table 2). These results suggest that diversification of agricultural systems using green manure produces a general rise in soil bacterial FAME, and the Gram+ bacteria are more efficient than the remaining microorganisms in their use of the nutrient supply generated by green manures. However, Zhao et al.^7^ reported that Gram− bacteria increased with increasing maize-straw input rates. These inconsistent results are probably attributable to differences in the green-manure species and the termination date. The abundance of actinomycetes was not affected by the green-manure treatments in this experiment (Table 2), because a variety of actinomycetes play important roles in degrading lignin and lignocelluloses, and a sufficient nutrient supply would not stimulate the growth of actinomycetes by degrading lignin^7^. The green-manure treatments resulted in a significantly greater relative abundance of saprophytic fungi compared to the no-green-manure treatment (Table 2). Most saprophytic soil microorganisms depend on organic C for their growth and metabolism^20^. In the current study, hairy vetch significantly decreased the relative abundance of the AMF biomarker while February orchid and rattail fescue revealed a significantly greater abundance of AMF compared to the no-green-manure treatment (Table 2). This may have been because hairy vetch increased nitrogen input into the soil due to its capacity to fix atmospheric N. Increased N availability negatively affects the abundance of mycorrhizal fungi^5^. When nutrients are readily available, plants allocate more photosynthates to shoots and leaves, and less to roots and mycorrhizal symbionts. AMF are growth promoting microorganisms and a large number studies have indicated that AMF can improve root traits^28–30^. In contrast, our results showed root morphological parameters were greater in hairy vetch treatment relative to the control in spite of having a lower AMF abundance (Table 2, Table 4). Similar results were also reported by some researchers that AMF had no or negative effect on the root length and the number of lateral root^31, 32^. These seemingly contradictory results suggest that the effect of AMF on root traits is complex, depending on AMF species and plant species. Further study is needed to investigate mechanisms of AMF involved in regulation of plant root system architecture.

In this study, green manure treatments increased tree height, ground diameter and crown breadth compared with the no-green-manure treatment. Similar results were also obtained on apple orchard^33, 34^. Green manures can improve soil nutrient contents, enzyme activities and SMB, thus improving walnut tree growth. Hairy vetch had the greatest effect on the growth of walnut tree than other green manure treatments, this may have been attributed to the highest level of TN and SOC found in the hairy vetch treatment. As a leguminous green manure, hairy vetch can introduce extra nitrogen into the soil via nitrogen fixation. These results are consistent with those obtained in a walnut orchard intercropped with *Vigna radiate*^35^. Root morphological parameters, play a decisive role in the availability of nutrients that are transported to the root surface mainly via diffusion^36^. Our results showed root length, root length density and root surface area were greater under green manure treatments relative to no green manure treatment, independent of the species used for the green manure. This indicated that green manures promoted the expansion of walnut root system and contributed to the growth and development of walnut root. Combining the results of present study, green manures improved the soil nutrient contents and promoted the growth and development of walnut root system, which ensured the walnut root could absorb enough nutrient for growth. Therefore, application of green manures was benefit to the vegetative growth of walnut tree.

Water content was significantly greater in soil under the flail relative to disk (Table 1), implying that soil water content was affected by the termination method. One of the major differences between flail and disk is the location where the green-manure residue is placed following termination. Green manure residues left on the soil surface vs. incorporated into the soil are thought to improve water infiltration, reduce soil surface, and therefore help maintain soil moisture. The flail treatment in the present study resulted in higher SOC and TN values compared to the disk treatment (Table 1). This is consistent with Mbuthia et al.^5^, who found that SOC and TN values increased under no-tillage, cover-cropped treatments compared to conventional practice treatments. The most possible reason is that no-tillage can enhanced the aggregate stability. Moreover, frequent tillage, which is conducted for rewetting of dry soil, caused SOC to become highly labile due to tillage disturbance^37^. The other possible reason is that termination method can affect soil N mineralization and nitrification. As suggested by Liang et al.^27^, N mineralization and nitrification were significantly greater under flail relative to disk. Our results suggested that tillage-dependent green manure treatments could significantly up-regulated soil N availability and therefore improve soil N fertility. The high P and mineral N concentrations observed under the flail treatment could be the effect of biological cycling attributed to the concurrent increase in soil organic matter under no tillage. Organic matter supplies nutrients as it is mineralized, and releases organic acids that increase P and N availability. There was no obvious difference in AK content between the flail and disk, which is consistent with the findings of Mbuthia et al.^5^. This may have been due to the great demand for K from the increased aboveground walnut tree biomass in the flail plots.

In this study, soil β-glucosidase, N-acetyl-glucosaminidase and acid phosphatase activities were significantly greater under flail relative to disk (Fig. 1). This result was likely due to the termination-method-associated changes in soil properties. This can be explained by the greater organic matter content and nutrient availability associated with no tillage, the lack of disrupted soil layers, and the less oxidizing environment, which stabilizes the pool of extracellular enzymes^4, 27^. The termination method had no significant effect on phenoloxidase activity in our study (Fig. 1D). Oxidative enzymes are involved in degrading recalcitrant compounds, such as lignin, during metabolic acquisition of nutrients. Fungi are one of sources of oxidative enzymes^23^, but fungi do not need to acquire N by degrading recalcitrant compounds under high soil fertility^7^. This could explain why there was no difference in oxidative enzyme activity between the flail and disk treatments.

SMB is often regulated by changes in environmental factors following tillage practices. Our results showed that flail was associated with greater SMB. No tillage with surface residue coverage reduces fluctuation in surface temperatures and moisture content and is conducive to soil aggregation and the accumulation of SOM, which are beneficial for the survival of soil microorganisms. A high MBC: MBN ratio indicates a greater proportion of fungi compared to bacteria. Reduced tillage promotes fungal growth and thus increases the fungal-to-bacteria biomass ratio, which is likely attributed to less disruption by fungal hyphal networks and increased soil moisture^38^. However, our results show that tillage did not have any significant effect on MBC, MBN or MBC: N (Fig. 2). These results may be explained by the sampling depth and sampling time. Several studies have shown that no tillage or reduced tillage is associated with greater SMB, but only in the top (about 5 cm) soil layer^20^.

In the present study, flail treatment resulted in significant shifts in the microbial community structure that was characterized by a greater relative abundance of FAME biomarkers associated with Gram+ bacteria, actinomycetes and mycorrhizal fungi (Table 2). The high abundance of Gram+ bacteria and actinomycetes has been related to anaerobic soil conditions. The abundance of actinomycetes decreases following green-manure termination with more intensive tillage implements, such as field disks^5, 39^. Mycorrhizae decrease under tillage due to disruptions in their hyphal network. Apart from the minimal disruption of hyphal networks, the abundance of fungi has been hypothesized to be greater under reduced tillage because their cell structural composition is comprised of chitin, which is more resistant to degradation and has been linked with high soil C sequestration^40^. Therefore, these physiological interactions may be the reason for the dominance of bacterial FAME in the soil of the flail treatments as observed in our research.

In the present study, vegetative growth of walnut tree was significantly greater under the flail method relative to the disk method. The reasons for the reduced growth in the disk treatment may be explained by the decrease in soil nutrient availability, enzyme activities, and changes in microbial community. Also, root damage caused by disking might have had a negative effect on tree growth. Disking is not a recommended practice because it decreased the content of soil nutrient and led to poor tree vigor that corresponded to reduced vegetative growth of walnut tree.

## Conclusions

Green manures altered soil nutrient contents, enzyme activities, SMB, microbial community structure and vegetative growth of walnut tree, and different types of green manure had different impacts. Compared to disking, green-manure termination with a flail resulted in significant shifts in the microbial community structure and soil-nutrient contents to conditions that favour C, N and P cycling, and that in turn improved the tree growth. Our results indicate that applying green manure benefits soil nutrient availability, enzyme activities, soil microbial biomass, microbial community structure and tree growth. According to the overall effects of green manures and their termination, the hairy vetch and flail treatment combination is recommended for walnut orchards in northern China. However, the long-term influences of green manure on soil biological status should be considered when selecting the types of green manure to be used.

## Materials and methods

### Experimental site and design

This study was conducted between 2016 and 2019 in Shunyi District, Beijing City, China, which has warm temperatures and a sub-humid continental monsoon climate. The experimental orchard was located in the walnut research field of the Beijing Academy of Forestry and Pomology Sciences, Beijing City, China (40°06′N, 116°93′E). Prior to the experiment’s establishment, the field was conducted on a winter wheat-summer maize rotation system. The average annual air temperature and precipitation between 2016 and 2019 were 11.2 °C and 620 mm, respectively, and two-thirds of the precipitation fell between June and September. The orchard soil type was brown soil. The main characteristics of the soil before the beginning of the experiment are shown in Table 5. The walnut tree cultivar grown in the orchard was ‘Lipin 1’. The age of the trees was 2 years, and the inter-row spacing × inter-tree spacing was 5 m × 4 m.

**Table 5:** Main characteristics of the soil (0–20 cm) before the start of the experiment.

Parameters (units)	
pH	8.09
total organic carbon (g kg^−1^)	11.5
total nitrogen (g kg^−1^)	0.86
total phosphorus (g kg^−1^)	1.03
total potassium (g kg^−1^)	20.41
available P (mg kg^−1^)	32.5
available K (mg kg^−1^)	199

The experimental design consisted of a split-plot design in a randomized complete block arrangement. The main plot (30 × 15 m) corresponded to the green-manure species. There were four main plots (30 m long × 15 m wide) for the green-manure treatments: no green manure, February orchid, hairy vetch and rattail fescue. The main plot was divided into two subplots (30 × 7.5 m), corresponding to the termination methods: flail and disk. The no-green-manure plot, in which clean tillage was used, was not split, as there was no green manure to terminate and compare. The green manures were cut and chopped using a 1.5-m flail mower 9G-1.5 (Yucheng HongRi Machinery Manufacturing Co., Ltd., Dezhou City, Shandong, P. R. China) for the flail treatment. The green manures were chopped and incorporated into approximate 15 cm of soil depth using a 2.5-m disk 1BZ-2.5 (Yucheng HongRi Machinery Manufacturing Co., Ltd.) for the disk treatment. The experiment consisted of seven treatments with three replicates each: no green manure, February orchid and flail, February orchid and disk, hairy vetch and flail, hairy vetch and disk, rattail fescue and flail, and rattail fescue and disk.

The green manures were planted in the 1.6-m-wide area between rows. Clean tillage was used between the trees. From 2016 to 2018, the green manures were sown in late September, grown over winter, and then terminated in May the next year. After termination, natural grass was implemented in the orchard. The sowing volumes were 15 kg/ha for February orchid and 30 kg/ha for hairy vetch. The same soil and plant management practices were used in all treatments.

### Soil sampling

In the third year of the experiment, soil samples were collected from the orchard on September 25, 2019. Six rhizosphere soils samples (0–20 cm depth) were collected from each plot, pooled together and mixed. Specifically, we dug along a main, coarse root that could be traced back to the trunk, and then picked out the branching fine roots (< 2 mm) and adhering soil. After shaking the fine roots gently, the soil adhering to the fine roots were carefully sampled with forceps and was defined as rhizosphere soil. Soil samples were transported to the laboratory in a cooler and passed through a 2-mm sieve. Each sample was divided into three subsamples. The first subsample was stored at 4 °C for the microorganism and enzyme analyses. The second subsample was air-dried and used to determine the soil chemical properties. The third subsample was stored at − 80 °C for analysis of FAME profiles.

### Characterization of soil chemical properties

Soil pH was determined based on air-dried samples using a 1:2.5 soil/water ratio. Gravimetric water content was determined by drying at 105 °C for 48 h. Total organic carbon (TOC) was determined by the wet oxidation method with KCrO_7_ at 170–180 °C, while total nitrogen (TN) was determined by the micro-Kjeldahl method. Soil mineral N was extracted from moist soil with 2 M KCl and quantified using a flow injection analyser (Westco Scientific Ltd, Brookfield, CT, USA). Soil available phosphorus (AP) was determined by the Olsen method. Soil available potassium (AK) was measured using a flame photometer after extraction with NH_4_OAc.

### Soil enzyme activities

Soil enzymes can be divided into two broad groups: (1) hydrolytic enzymes (β-glucosidase, N-acetyl-glucosaminidase, acid phosphatase) responsible for the acquisition of C, N, and phosphorus (P) to support primary metabolism; and (2) oxidative enzyme (phenoloxidase), which degrade poor-quality, chenmically coplex compounds like lignin in comtabolic acquisition of nutrients^7, 41^. The activities of β-glucosidase, N-acetyl-glucosaminidase, acid phosphatase and phenoloxidase were measured using the methods of DeForest^42^ and Saiya-Cork et al.^43^. We conducted assays using 96-well microtiter plates, with eight replicate wells per sample per assay. The analysis involved eight replicate wells for each blank, a negative control, and a quench standard. For the hydrolytic enzyme assay, the buffer, sample suspension, 10 μM of the references and 200 μM of the substrates (4-methyl-umbelliferone or 7-amino-4-methylocumarin) were dispensed into the wells of black 96-well microplates. The microplates were covered and incubated in the dark at 20 °C for 0.5 h for N-acetyl-glucosaminidase and acid phosphatise, 2 h for, β-glucosidase. Fluorescence was measured using a microplate fluorometer (SynergyH4 MLFPT BioTek, Winooski, VT, USA) with 365-nm excitation and 450-nm emission filters. In the plate reader parameters, the number of flashes was set to 3, namely the fluorescence intensity per well was measured three times. Phenol oxidase was measured in a clear 96-well microplate using the substrate L-3, 4-dihydroxyphenylalanine. The dispensed volume and the order of buffer, sample suspension and 25-mM substrate were the same as for the fluorometric enzymes. The microplates were covered and incubated at 20 °C for 20 h in the dark, after which activity was assayed using a microplate fluorometer. The enzyme activities were expressed in nmol h^−1^ g^−1^.

### Characterization of soil microbial biomass and structure

Soil microbial biomass C (MBC) and N (MBN) were determined by the chloroform fumigation extraction method^5^. Briefly, 10 g of oven-dried equivalent samples were fumigated in the dark for 48 h after which C and N were extracted from the fumigated samples with 0.5 M K_2_SO_4_. Non-fumigated soil samples were subjected to the same 0.5 M K_2_SO_4_ extraction procedure. Total dissolved organic C (DOC) and total extractable N were measured with a C analyser (Model TOC/TN; Shimadzu, Tokyo, Japan). MBC and MBN were estimated by calculating the difference between the fumigated and non-fumigated samples, using a correction factor (kc: 0.35).

Soil microbial community composition was characterized using the EL-FAME analytical method^44^. The extracted fatty acids were isolated on a silica-bonded phase column and transesterified to FAMEs using a mild alkaline methanolysis reaction. The FAMEs were quantified using gas chromatography (N6890, Agilent Technologies, Palo Alto, CA, USA) and identified using a MIDI SHERLOCK microbial identification system (Version 4.5, MIDI, Inc., Newark, DE, USA). Nonadecanoic acid methyl ester (C19:0) was used as an internal standard to quantify the FAMEs. Total FAME concentration was expressed in units of nmol g^−1^ soil. The abundance of individual FAMEs was indicated by their % mol abundance in each sample. Selected FAMEs were used as microbial markers based on previously published data^45^. i15:0, a15:0, i16:0, i17:0 and a17:0 were used as markers for Gram+ bacteria, whereas 16:1ω7c, 16:1ω9c, cy17:0 and cy19:0 were used as markers for Gram− bacteria. 10Me16:0, 10Me17:0 and 10Me18:0 were used to represent actinomycetes biomarkers. Fungal indicators included saprophytic fungi (18:1ω9c, 18:2ω6c and 18:3ω6c) and arbuscular mycorrhizal fungi (AMF) (16:1ω5c). The bacterial sum was calculated based on the summation of Gram +, Gram−, and actinomycetes biomarkers; fungal sums were calculated using the saprophytic and AMF fungal markers. The fungal/bacteria (F: B) ratio was calculated by dividing the fungal sum by the bacterial sum.

### Characterization of plant growth parameters

Experimental design was a complete randomized block, with three replications and three plants for block. Tree height, ground diameter and crown breadth were recorded with the help of a measuring tape and a vernier calliper on September 25, 2019. The ground diameter was the diameter near the ground surface. The crown breadth *was* calculated as the average of two values measured along two perpendicular directions. At the same time, the roots of walnut were collected. The root samples were washed with tap water, and then were spread out on a transparent, water-filled tray (15 cm × 25 cm) and scanned with the WinRhizo root measuring system (WinRhizo, Régent Instruments Inc., Quebec, Canada). Root morphological variables (surface area, length and length density) were obtained by means of image analysis software (WinRhizo Reg 2005c).

### Data analysis

Data were analyzed using a two-way analysis of variance (ANOVA) based on a general linear model. The fixed factors were green manure and tillage. Replication was considered to be a random factor. When the main effect was significant, multiple comparisons of means among treatments were conducted with the least significant difference (LSD) test. When confirming a statistically significant *P* value, the Fisher test (*P* < 0.05) was used for comparison. A principal component analysis (PCA) was performed on a correlation matrix to distinguish treatment separation of the microbial community structure based on the FAMEs. Analysis of variance was performed using SPSS 13.0 (SPSS Inc., Chicago, IL, USA). Canoco 4.5 (MicrocomputerPower, Ithaca, USA) was used to run PCA. The histogram was created using Microsoft Excel 2010 (Microsoft, Redmond, Washington, USA).

## Supplementary Information

Supplementary Information.
