# Supplementary material for: Effects of green-manure and tillage management on soil microbial community composition, nutrients and tree growth in a walnut orchard
Source: Sci Rep. 2021 Aug 19;11:16882. doi: 10.1038/s41598-021-96472-8 (PMC8377041; doi:10.1038/s41598-021-96472-8)
Supplement: Supplementary file 1 — Supplementary Information. [file 41598_2021_96472_MOESM1_ESM.pdf]

Effects of green-manure and tillage management on soil microbial community composition, nutrients and tree growth in a walnut orchard

Ningguang Dong[†], Guanglong Hu[†], Yunqi Zhang, Jianxun Qi, Yonghao Chen, Yanbin Hao*

Beijing Academy of Forestry and Pomology Sciences, Beijing Academy of Agriculture and Forestry Sciences, Beijing 100093, P.R. China

* Corresponding author: 32521990@qq.com; walnut@baafs.net.cn

[†] These authors contributed equally to this work

Table S1. The P-value of ANOVA corresponding to Table 1.

**Treatment**	pH	Water content	Organic C	Total N	Mineral N	Available P	Available K
**Green manure**	0.165	0.036	0.022	0.010	0.028	0.001	0.002
**Tillage**	0.523	0.029	0.039	0.027	0.037	<0.0001	0.013
**Repeat**	0.953	0.909	0.196	0.620	0.478	0.420	0.910
**Green manure ×Tillage**	0.438	0.558	0.762	0.431	0.533	0.322	0.088

Table S2. The P-value of ANOVA corresponding to Figure 1.

**Treatment**	β-glucosidase	N-acetyl-glucosaminidase	Acid phosphatise	Phenoloxidase
**Green manure**	0.000	0.027	0.000	0.003
**Tillage**	0.024	0.019	0.010	0.206
**Repeat**	0.472	0.741	0.214	0.083
**Green manure ×Tillage**	0.399	0.498	0.189	0.232

Table S3. The P-value of ANOVA corresponding to Figure 2.

**Treatment**	MBC	MBN	MBC/MBN
**Green manure**	0.006	0.002	0.001
**Tillage**	0.959	0.630	0.903
**Repeat**	0.143	0.051	0.293
**Green manure ×Tillage**	0.343	0.456	0.857

Table S4. The P-value of ANOVA corresponding to Table 2.

**Treatment**	Total FAME	bacterial			Total bacteria	Fungi		Total fungi	G+/G-	F/B
		G+	G-	actinomycetes		saprophytic	AMF			
**Green manure**	0.015	0.004	0.979	0.025	0.029	0.006	0.004	0.031	0.032	0.596
**Tillage**	0.366	0.001	0.884	0.007	0.563	0.000	0.002	<0.0001	0.004	0.034
**Repeat**	0.058	0.390	0.082	0.432	0.054	0.061	0.085	0.243	0.941	0.657
**Green manure ×Tillage**	0.591	0.173	0.547	0.625	0.741	0.497	0.706	0.411	0.968	0.640

Table S5. The P-value of ANOVA corresponding to Table 3.

**Treatment**	Tree height	Ground diameter	Crown breadth
**Green manure**	0.006	0.022	<0.0001
**Tillage**	0.001	0.003	0.000
**Repeat**	0.054	0.059	0.052
**Green manure ×Tillage**	0.648	0.405	0.741

Table S6. The P-value of ANOVA corresponding to Table 4.

**Treatment**	Root length	Root length density	Root surface area
**Green manure**	0.011	0.000	0.004
**Tillage**	0.028	0.001	0.023
**Repeat**	0.588	0.770	0.723
**Green manure ×Tillage**	0.162	0.208	0.158
